# Supplementary material for: Saliency-enhanced infrared and visible image fusion via sub-window variance filter and weighted least squares optimization
Source: PLoS One. 2025 Jul 7;20(7):e0323285. doi: 10.1371/journal.pone.0323285 (PMC12233290; doi:10.1371/journal.pone.0323285)
Supplement: Attachment — Submitted filename: Response to Reviewers.pdf [file pone.0323285.s003.pdf]

Responses to Reviewers

Note: For easy identification, additions or modifications are marked ***in blue text in the revised manuscript.***

Responses to Reviewer 1's comments:

Reviewer #1: The article was reviewed under the title "Infrared and visible image fusion method based on sub-window variance filter and weighted least squares optimization". Overall, the focus of the study is good. However, I would like to offer several recommendations that authors may find useful in the process of revising their manuscript:

1- The abstract is very general, and it is necessary to provide more details in the abstract, given the variety of topics discussed.

Response: We are very grateful for the suggestions provided by the reviewers.

In accordance with the reviewers' suggestions, we have carefully revised the abstract. In the revised abstract, we have added more technical details, such as the SVF decomposition, WLSO-based weight map computation, and pixel-level reconstruction process. Additionally, we included the limitations of the method (e.g., potential challenges in handling highly complex scenes) and future directions for improvement. The revised abstract is as follows:

"This paper proposes a novel method for infrared and visible image fusion (IVIF) to address the limitations of existing techniques in enhancing salient features and improving visual clarity. The method employs a sub-window variance filter (SVF) based decomposition technique to separate salient features and texture details into distinct band layers. A saliency map measurement scheme based on weighted least squares optimization (WLSO) is then designed to compute weight maps, enhancing the visibility of important features. Finally, pixel-level summation is used for feature map reconstruction, producing high-quality fused images. Experiments on three public datasets demonstrate that our method outperforms nine state-of-the-art fusion techniques in both qualitative and quantitative evaluations, particularly in salient target highlighting and texture detail preservation. Unlike deep learning-based approaches, our method does not require large-scale training datasets, reducing dependence on ground truth and avoiding fused image distortion. Limitations include potential challenges in handling highly complex scenes, which will be addressed in future work by exploring adaptive parameter optimization and integration with deep learning frameworks."

Thank you again for your constructive comments.

2- Considering the classification of Related works into two categories: conventional methods and deep learning, it is better to examine more of the activities carried out in order to compare and show the strengths.

Response: We sincerely thank the reviewer for their valuable suggestion.

According to the reviewers' suggestions, we have incorporated a more extensive discussion of existing advanced methods in the related work. The added content is as follows:

Regarding conventional methods: "Liu et al. [1] have extracted infrared target features and texture details from the original images by performing coarse-scale decomposition on infrared images and fine-scale decomposition on visible images, respectively. To obtain effective information from the source images, Li et al. [2] proposed a fusion method based on latent low-rank representation. However, this method faces significant computational efficiency issues due to the extensive mathematical calculations required to obtain the optimal solution."

Regarding deep learning-based methods: "PMGI utilizes multi-gradient information to guide the image fusion process and employs CNN to extract and fuse multi-scale features [3]. Building upon NestFuse, Li et al. [4] proposed a learnable residual fusion network to replace manually designed fusion strategies, effectively addressing the issue of insufficient information utilization. Xing et al. [5] introduced a compressed fusion network named CFNet, which incorporates the idea of image compression based on variational autoencoders to achieve joint optimization of image fusion and data compression. To tackle the problem of global information loss, Wu et al. [6] proposed a globally aware generative adversarial fusion network and introduced differential image loss to further constrain the generator to learn important information from the source images."

We believe these revisions provide a more comprehensive and balanced discussion of related works. Thank you for your constructive feedback, and we hope the revised version meets your expectations.

References:

[1] Liu Y, Dong L, Ren W, et al. Multi-scale saliency measure and orthogonal space for visible and infrared image fusion. Infrared Physics & Technology, 2021, 118: 103916.

[2] Li H, Wu X J. Infrared and visible image fusion using latent low-rank representation. arXiv preprint arXiv:1804.08992, 2018.

[3] Zhang H, Xu H, Xiao Y, et al. Rethinking the image fusion: A fast unified image fusion network based on proportional maintenance of gradient and intensity. Proceedings of the AAAI conference on artificial intelligence. 2020, 34(07): 12797-12804.

[4] Li H, Wu X J, Kittler J. RFN-Nest: An end-to-end residual fusion network for infrared and visible images. Information Fusion, 2021, 73: 72-86.

[5] Xing M, Liu G, Tang H, et al. CFNet: An infrared and visible image compression fusion network. Pattern Recognition, 2024, 156: 110774.
[6] Wu J, Liu G, Wang X, et al. GAN-GA: infrared and visible image fusion generative adversarial network based on global awareness. Applied Intelligence, 2024: 1-21.

3- Considering the superiority of the model, if the challenges of previous research are examined, it can enrich the effectiveness of the article.

Response: We greatly appreciate the suggestions made by the reviewers.

We sincerely thank the reviewer for their valuable suggestion to examine the challenges of previous research and highlight the superiority of our model. In response to this comment, we have added a detailed discussion of the limitations of existing methods and how our proposed method addresses these challenges. This discussion has been incorporated into the introduction and experimental sections of the manuscript to provide a clearer context for our work and emphasize its contributions.

The added content is marked with **blue text in the revised manuscript (**Introduction and experimental sections**)** for easy identification.

We hope this change meets your expectations. Thanks again for the advice you've provided.

4- The article states that the weight factor (CK) is divided into 4 sub-windows. Are the 4 sub-windows specified by the authors or is it a fixed value?

Response: We sincerely thank the reviewer for this question.

The division of the filtering region into four sub-windows (A, B, C, and D) is a design choice specified by the authors. This division is based on the spatial structure of the image and the need to capture local variance information effectively. The four sub-windows are created by dividing the filtering region WW into four equal-sized quadrants, which allows for a balanced representation of local edge statistics in different directions (e.g., horizontal, vertical, and diagonal). This approach ensures that the filter can adaptively preserve edge features while smoothing homogeneous regions.

Thanks again for your valuable suggestions.

5- If the authors chose 4 sub-windows, what was the reason for choosing this value?

Response: We appreciate the reviewer's interest in the rationale behind our choice of four sub-windows.

The selection of four sub-windows is based on the following considerations:

(1) **Balanced Representation of Local Variance**: Dividing the filtering region into four sub-windows provides a balanced representation of local variance in different spatial directions (e.g., horizontal, vertical, and diagonal). This allows the filter to

effectively capture edge and texture information while avoiding over-smoothing in regions with significant structural variations.

(2) **Computational Efficiency**: Using four sub-windows strikes a balance between computational complexity and the accuracy of local variance estimation. A smaller number of sub-windows (e.g., two) may not adequately capture the spatial diversity of local variance, while a larger number (e.g., eight) would increase computational overhead without significantly improving performance.

(3) **Consistency with Prior Work**: The choice of four sub-windows is also supported by prior work in edge-preserving filters, where a similar division has been shown to effectively balance edge preservation and noise reduction [42].

In addition, we have supplemented relevant explanations in Section 3.2. The added content is as follows:

"… This division is based on the need to capture local variance information effectively in different spatial directions (e.g., horizontal, vertical, and diagonal). The choice of four sub-windows provides a balanced representation of local edge statistics, ensuring that the filter can adaptively preserve edge features while smoothing homogeneous regions. Additionally, this choice strikes a balance between computational efficiency and the accuracy of local variance estimation. …"

Thank you again for your constructive comments.

6- Although the evaluation criteria used are well-known, it is better to include their full text in a footnote the first time their summary is used in the text of the article.

Response: We would like to express our sincere gratitude to the reviewers for their constructive comments.

In accordance with the reviewers' suggestions, we have revised the relevant content, and the updated version is as follows:

"In the quantitative experiments, six pivotal evaluation metrics were chosen to assess the fusion performance comprehensively, including spatial frequency (SF) [1], mean squared error (MSE) [2], correlation coefficient (CC) [3], sum of the correlations of differences (SCD) [4], structural similarity index measure (SSIM) [5], and peak signal to noise ratio (PSNR) [6]."

References:

[1] Eskicioglu A M, Fisher P S. Image quality measures and their performance[J]. IEEE Transactions on communications, 1995, 43(12): 2959-2965.
[2] Bauer E, Kohavi R. An empirical comparison of voting classification algorithms: bagging, boosting, and variants. Mach Learn 1999;36:105–39.
[3] Deshmukh M, Bhosale U. Image fusion and image quality assessment of fused images[J]. International Journal of Image Processing (IJIP), 2010, 4(5): 484.

[4] Aslantas V, Bendes E. A new image quality metric for image fusion: The sum of the correlations of differences[J]. Aeu-international Journal of electronics and communications, 2015, 69(12): 1890-1896.
[5] Wang Z, Bovik A C, Sheikh H R, et al. Image quality assessment: from error visibility to structural similarity[J]. IEEE transactions on image processing, 2004, 13(4): 600-612.
[6] Jin X, Jiang Q, Yao S, et al. A survey of infrared and visual image fusion methods[J]. Infrared Physics & Technology, 2017, 85: 478-501.

Thank you again for your constructive comments.

7- In the tables where the evaluation criteria obtained are compared with other studies, in some cases the criteria obtained by other researchers have a slight advantage, but the authors have stated that their model has superiority.

Response: We sincerely appreciate the reviewer's careful observation regarding the quantitative comparison results.

While it is true that some individual metrics in the tables show slight advantages for other methods, our proposed method demonstrates overall superiority across multiple datasets and evaluation criteria. Specifically:

(1) Our method achieves the **best or second-best results** in the majority of metrics across all three datasets (TNO, RoadScene, and LLVIP). For example:

<1> In Table 1 (TNO dataset), our method ranks **first in 5 out of 6 metrics** (SF, MSE, SCD, SSIM and PSNR) and **second in CC**.

<2> In Table 2 (RoadScene dataset), our method achieves the **best results in MSE and PSNR**, with competitive performance in other metrics.

<3> In Table 3 (LLVIP dataset), our method ranks **second in SF, MSE, CC, SSIM and PSNR**.

(2) While some methods may excel in one or two specific metrics, they often underperform in others.

(3) Our method's superiority lies not only in quantitative metrics but also in its ability to **enhance salient features** and **preserve texture details**, as highlighted in the qualitative results and visual comparisons in the manuscript.

In summary, while other methods may be slightly better on some individual metrics, the overall performance of our method on multiple datasets and assessment metrics proves our advantage. Thank you for your valuable comments, which helped us improve the clarity and accuracy of our presentation.

Responses to Reviewer 2's comments:

Reviewer #2:

1- The title should be improved.

Response: Thank you for your valuable feedback regarding the title of our manuscript.

We have revised the title to enhance its clarity and readability. The new title is:

"Saliency-Enhanced Infrared and Visible Image Fusion via Sub-Window Variance Filter and Weighted Least Squares Optimization"

We believe this modification better highlights the core techniques used in our image fusion method and provides a clearer understanding of the paper's focus. We hope this change meets your expectations. Thank you again for your constructive comments.

2- The objectives and the rationale of the study are recommended to be clearly stated.

Response: We sincerely thank the reviewer for this suggestion.

In the revised introduction, we have explicitly stated the objectives of our study: to address the limitations of existing fusion methods, particularly the inadequate enhancement of salient features and the reliance on large-scale training datasets.

The newly added relevant content in our introduction is as follows:

"The primary objective of this study is to address the limitations of existing fusion methods, particularly the inadequate enhancement of salient features and the reliance on large-scale training datasets. Our proposed method aims to effectively separate and highlight salient features while preserving texture details, without requiring extensive training data, thereby improving the quality and interpretability of infrared and visible image fusion."

Thanks again for the advice you've provided.

3- The concluding remarks of the abstract are not well-written. It's merely the repetition of the objectives and title of the manuscript. Please add method limitations and justification to the abstract.

Response: We greatly appreciate the suggestions made by the reviewers.

Following the reviewers' advice, we have meticulously revised the abstract. The revised conclusion avoids repetition and emphasizes the advantages of the method. It clearly outlines the limitations and future research directions. The revised abstract is as follows:

"This paper proposes a novel method for infrared and visible image fusion (IVIF) to address the limitations of existing techniques in enhancing salient features and improving visual clarity. The method employs a sub-window variance filter (SVF) based decomposition technique to separate salient features and texture details into

distinct band layers. A saliency map measurement scheme based on weighted least squares optimization (WLSO) is then designed to compute weight maps, enhancing the visibility of important features. Finally, pixel-level summation is used for feature map reconstruction, producing high-quality fused images. Experiments on three public datasets demonstrate that our method outperforms nine state-of-the-art fusion techniques in both qualitative and quantitative evaluations, particularly in salient target highlighting and texture detail preservation. Unlike deep learning-based approaches, our method does not require large-scale training datasets, reducing dependence on ground truth and avoiding fused image distortion. Limitations include potential challenges in handling highly complex scenes, which will be addressed in future work by exploring adaptive parameter optimization and integration with deep learning frameworks."

Thanks again for your valuable suggestions.

4- The innovation of using this study is not very clear. I do not see a clear reason that this study can perform better than others. Why did the authors choose the method for this study?

Response: We appreciate the reviewer's comment.

In the revised introduction, we have highlighted the innovation of our study, which lies in the combination of the sub-window variance filter (SVF) for decomposition and the weighted least squares optimization (WLSO) for saliency map measurement. This combination allows our method to effectively separate and enhance salient features while preserving texture details, outperforming existing methods in both qualitative and quantitative evaluations.

The newly added relevant content is as follows:

"In short, the innovation and advantage of our study lies in the combination of the SVF for decomposition and the WLSO for saliency map measurement. This combination allows our method to effectively separate and enhance salient features while preserving texture details, addressing the limitations of existing methods that either neglect salient feature enhancement or rely on large-scale training datasets."

We hope that the revisions we have made will meet your satisfaction.

5- The necessity & novelty of the manuscript should be presented and stressed in the "Introduction" section.

Response: Thank you for this suggestion.

In the revised introduction, we have strengthened the discussion of the necessity and novelty of our work. We have explicitly stated the gaps in existing methods, such as the lack of salient feature enhancement and the reliance on large-scale training datasets, and explained how our method addresses these gaps. We have also emphasized the

novelty of our approach, particularly the use of SVF and WLSO, which are not commonly combined in existing fusion methods.

Therefore, we have dedicated a paragraph in the introduction to summarize our innovations and advantages. The added content is as follows:

"In short, the innovation and advantage of our study lies in the combination of the SVF for decomposition and the WLSO for saliency map measurement. This combination allows our method to effectively separate and enhance salient features while preserving texture details, addressing the limitations of existing methods that either neglect salient feature enhancement or rely on large-scale training datasets."

Thank you for your constructive feedback, and we hope the revised version meets your expectations.

6- The application/theory/method/study reported is not in sufficient detail to allow for its replicability and/or reproducibility. Therefore, it is suggested to make it clear to show all steps to build the model.

Response: We sincerely thank the reviewer for this suggestion.

We have thoroughly revised the methodology section to ensure that all steps of our proposed method are clearly described and detailed enough to allow for replicability and reproducibility. Specifically, we have added:

(1) Detailed Steps for Image Decomposition:

We have elaborated on the image decomposition process using the Sub-Window Variance Filter (SVF). Each step, from dividing the filtering region into sub-windows to calculating the weight coefficient C_k, is now clearly explained with corresponding equations (Eqs. 8, 10, 11, and 12). This ensures that readers can precisely follow the procedure to decompose the source images into base and detail layers.

(2) Comprehensive Fusion Strategy:

The fusion strategy section has been expanded to include detailed descriptions of how the visual saliency maps are computed (Eq. 14) and optimized using Weighted Least Squares Optimization (WLSO) (Eqs. 15 and 19). We have also explicitly shown how the final weight coefficients are calculated (Eq. 4) and applied to fuse the base and detail layers (Eqs. 5, 6, and 7).

(3) Overall Framework Clarification:

In the overall overview section (Section 3.1), we have systematically outlined each step of our method, from image decomposition to the final fusion result. Each step is supported by clear equations and explanations, making it easier for readers to understand and reproduce the entire pipeline.

We believe that these revisions address your concerns regarding the clarity and replicability of our method. We have ensured that every step is thoroughly explained,

and the necessary equations are provided to allow other researchers to implement and reproduce our work. The added content is marked with **blue text in the revised manuscript** for easy identification.

Thanks again for the advice you've provided.

7- The problem statement and gap study are not clear.

Response: We appreciate the reviewer's feedback.

In the revised introduction, we have clarified the problem statement and the gaps in existing research. We have explicitly stated the limitations of current fusion methods, such as the inadequate enhancement of salient features and the reliance on large-scale training datasets, and explained how our method addresses these limitations. This provides a clearer context for our work and highlights its significance.

The changes made in the introduction are as follows:

"…Although most existing algorithms produce relatively favorable fusion results, certain limitations remain to be addressed. 1) many traditional methods, such as those based on MST or SR, rely on handcrafted fusion rules that may not effectively enhance salient features while preserving texture details. 2) Existing fusion methods obtain satisfactory fused images by designing complex fusion networks, however, the enhancement of salient features is neglected while ensuring the richness of feature information. As shown in Fig. 1, both SDNet and ICAFusion result in a fused image that contains complete information, but falls short in highlighting the salient features of thermal radiation, as shown in the purple box. 3) Deep learning-driven fusion methods often require extensive datasets of high-quality natural images to train networks, aiming to make the fusion model have excellent fusion performance and robustness. These limitations highlight the need for a new approach that can effectively enhance salient features without relying on large-scale training data…".

Thanks again for your valuable suggestions.

8- The method is not clear. Therefore, it must be shown and clarified to show all steps.

Response: We appreciate the reviewer's feedback regarding the clarity of our method.

Based on reviewers' suggestions, we have restructured and clarified the method section to ensure that all steps are clearly explained. Key additions include:

(1) Step-by-Step Methodology:

We have restructured the methodology section to present a clear, step-by-step breakdown of our approach. Each stage of the method, including image decomposition, visual saliency map computation, weight optimization, and fusion of base and detail layers, is now clearly delineated with detailed descriptions and supporting equations.

(2) Enhanced Explanation of Key Components:

Image Decomposition: We have provided a detailed explanation of how the Sub-Window Variance Filter (SVF) works, including the division of the filtering region into sub-windows and the calculation of the weight coefficient C_k (Eqs. 8, 10, 11, and 12).

Visual Saliency Map and Weight Optimization: The computation of visual saliency maps (Eq. 14) and the optimization process using Weighted Least Squares Optimization (WLSO) (Eqs. 15 and 19) are now clearly described.

Final Fusion Process: The steps to obtain the final fused image, including the calculation of weight coefficients (Eq. 4) and the fusion of base and detail layers (Eqs. 5, 6, and 7), are explicitly shown.

(3) Improved Readability:

We have added clear headings and subheadings to organize the methodology section better. This makes it easier for readers to follow the flow of our method and understand each component's role in the overall pipeline.

By providing a detailed and structured presentation of our method, we aim to enhance its clarity and ensure that readers can easily follow and understand each step. We sincerely hope that these revisions meet your expectations and address the concerns raised. We are confident that our method is now presented in a clear and detailed manner, facilitating both understanding and reproducibility.

Thank you again for your valuable feedback, which has helped us improve the quality of our manuscript.

9- The interpretation of results and study conclusions are not supported by providing the reasons behind why they show that. Therefore, it is recommended to deepen the discussion.

Response: Thank you for your valuable feedback.

We acknowledge that the interpretation of the results and study conclusions in the original manuscript could be further deepened by providing more detailed explanations for the observed phenomena. We have revised the relevant sections to include a more thorough discussion of the reasons behind the results.

The added content is marked with **blue text in the revised manuscript** for easy identification.

We hope these revisions address the concerns raised by the reviewer. If further clarification is needed, we are happy to provide additional details. Thank you for your valuable feedback, which has significantly improved the depth and clarity of our manuscript.

10- It is recommended to emphasize the strengths of the study clearly.

Response: We thank the reviewer for this suggestion.

In the revised introduction, we have added a dedicated paragraph summarizing the strengths of our study. These include the novel combination of SVF and WLSO, the ability to enhance salient features while preserving texture details, and the independence from large-scale training datasets. We have also highlighted the superior performance of our method in both qualitative and quantitative evaluations, as demonstrated by the experimental results.

The newly added relevant content is as follows:

“The innovation and advantage of our study lies in the combination of the sub-window variance filter (SVF) for decomposition and the weighted least squares optimization (WLSO) for saliency map measurement. This combination allows our method to effectively separate and enhance salient features while preserving texture details, addressing the limitations of existing methods that either neglect salient feature enhancement or rely on large-scale training datasets.”

Once again, we sincerely thank you for your valuable feedback.

11- The limitations of the study should be stated.

Response: We sincerely thank the reviewer for their valuable suggestion to include a discussion of the limitations of our study.

In response, we have appended a dedicated section following the conclusion to delineate the limitations of our proposed method and to outline potential areas for improvement. The additional content is as follows:

Limitations and Future Work:

“Despite the promising results achieved by our proposed method, several limitations should be noted. First, while our method performs well on standard datasets, it may face challenges in highly complex scenes where the distinction between salient targets and background textures is less clear. Future work could explore adaptive parameter optimization to improve performance in such scenarios. Second, the applicability of our method to other fusion tasks, such as medical image fusion or multi-spectral fusion, has not been fully explored. Future studies will investigate the generalization capability of our approach across different domains.”

Thank you for your constructive feedback, which has helped us improve the transparency and completeness of our manuscript.

12- The manuscript structure, flow, or writing needs some improvements.

Response: We sincerely appreciate the reviewer's feedback regarding the structure, flow, and writing of the manuscript.

We acknowledge that there is room for improvement in these aspects, and we have taken the following steps to address the concerns:

We have reorganized the manuscript to ensure a logical flow of ideas. Specifically, we have refined the introduction to better highlight the motivation and contributions of our work, and we have restructured the methodology section to provide a clearer step-by-step explanation of our proposed method. We have carefully revised the language to improve clarity, conciseness, and precision. We have also addressed grammatical issues and improved the overall readability of the text.

We believe these revisions have significantly improved the structure, flow, and writing of the manuscript, making it more engaging and easier to follow. Thank you for your constructive feedback, which has helped us enhance the quality of our work. We hope the revised version meets your expectations.

13- The manuscript is benefit from language editing. The English of the paper is readable; however, I would suggest the authors to have it checked preferably by a native English-speaking person to avoid any mistakes.

Response: We sincerely thank the reviewer for their valuable suggestion regarding language editing.

We acknowledge that while the English in the manuscript is readable, there is room for improvement to ensure clarity, precision, and fluency. In response to this feedback, we have carefully revised the manuscript to address any remaining issues and ensure that the language is clear, concise, and academically appropriate.

We believe these revisions have significantly enhanced the quality of the language in the manuscript, ensuring that it meets the high standards expected for publication. Thank you for your constructive feedback, which has helped us improve the readability and professionalism of our work.

14- I noticed that the conclusion section tends to repeat the abstract and results. The conclusion paragraph should be short, impactful, and direct the reader to this research's next steps and opportunities.

Response: We thank the reviewers for their valuable suggestions.

Based on the reviewers' recommendations, we have modified the conclusion. The revised conclusion is more concise and directly highlights the contributions. In addition, we have appended a dedicated section following the conclusion to delineate the limitations of our proposed method and to outline potential areas for improvement. It avoids redundancy and provides readers with a clear direction for further exploration. The revised conclusion and the additional content are as follows:

"In this paper, we proposed a novel IVIF method based on SVF and WLSO, effectively separating salient features and texture details while enhancing their visibility in fused images. Experimental results demonstrate superior performance over nine advanced methods, particularly in target highlighting and detail preservation. The method's independence from large-scale training datasets is a significant advantage, reducing computational costs and avoiding distortion issues.

Limitations and Future Work:

Despite the promising results achieved by our proposed method, several limitations should be noted. First, while our method performs well on standard datasets, it may face challenges in highly complex scenes where the distinction between salient targets and background textures is less clear. Future work could explore adaptive parameter optimization to improve performance in such scenarios. Second, the applicability of our method to other fusion tasks, such as medical image fusion or multi-spectral fusion, has not been fully explored. Future studies will investigate the generalization capability of our approach across different domains."

15- It will be nice to add some new references to show that your study is updated, such as: Zhou, Zhanxin, and Ruibo Wu. "Stock Price Prediction Model Based on Convolutional Neural Networks." Journal of Industrial Engineering and Applied Science 2.4 (2024): 1-7; Alakbari, Fahd Saeed, et al. "Prediction of critical total drawdown in sand production from gas wells: Machine learning approach." The Canadian Journal of Chemical Engineering 101.5 (2023): 2493-2509.; Alakbari, Fahd Saeed, et al. "Deep learning approach for robust prediction of reservoir bubble point pressure." ACS omega 6.33 (2021): 21499-21513.; Alakbari, Fahd Saeed, et al. "A gated recurrent unit model to predict Poisson's ratio using deep learning." Journal of Rock Mechanics and Geotechnical Engineering 16.1 (2024): 123-135; Zhou, Zhanxin, and Ruibo Wu. "Stock Price Prediction Model Based on Convolutional Neural Networks." Journal of Industrial Engineering and Applied Science 2.4 (2024): 1-7; Wu, Ruibo, Tao Zhang,

Response: We extend our heartfelt gratitude to the reviewers for their insightful suggestions.

In response to the reviewers' recommendations, we have incorporated references to the following research papers in the revised manuscript.

[1] Zhou Z, Wu R. Stock Price Prediction Model Based on Convolutional Neural Networks. Journal of Industrial Engineering and Applied Science, 2024, 2(4): 1-7.

[2] Alakbari F S, Mohyaldinn M E, Ayoub M A, et al. Prediction of critical total drawdown in sand production from gas wells: Machine learning approach. The Canadian Journal of Chemical Engineering, 2023, 101(5): 2493-2509.

[3] Alakbari F S, Mohyaldinn M E, Ayoub M A, et al. Deep learning approach for robust prediction of reservoir bubble point pressure. ACS omega, 2021, 6(33): 21499-21513.

[4] Alakbari F S, Mohyaldinn M E, Ayoub M A, et al. A gated recurrent unit model to predict Poisson's ratio using deep learning. Journal of Rock Mechanics and Geotechnical Engineering, 2024, 16(1): 123-135.

Responses to Reviewer 3's comments:

Reviewer #3:

1. In the process of introducing the sub - window variance filter (SVF) and weighted least squares optimization (WLSO), the following aspects need to be improved:

- Provide a more detailed explanation of the derivation steps of key formulas to help readers understand their mathematical principles. For some assumptions, clarify their rationality and basis.

Response: We thank the reviewer for pointing out the need for a more detailed explanation of the mathematical derivations and assumptions. Below, we provide additional clarifications for the key formulas and assumptions in our method:

(1) Sub-window Variance Filter (SVF):

The SVF is designed to separate an image into base and detail layers by computing local variance statistics. The weight coefficient C_k is calculated based on the ratio of the maximum and minimum variance values in the filtering region W. This approach ensures that edge features are preserved while smoothing homogeneous regions.

The assumption of dividing the filtering region into four sub-windows is based on the need to capture local variance information in different spatial directions (e.g., horizontal, vertical, and diagonal). This choice is empirically validated and provides a balanced representation of local edge statistics.

(2) Visual Saliency Maping (VSM):

The saliency map $S(i)$ is computed as the sum of absolute differences between the intensity of pixel ii and all other pixels in the image. This approach highlights regions with high contrast, which are typically more salient.

The normalization of $S(i)$ to the range [0,1] ensures that the saliency values are comparable across different images.

(3) Weighted Least Squares Optimization (WLSO):

The optimization function in Eq. (15) aims to minimize the difference between the saliency map S and the weight map W, while penalizing large gradients in W. This ensures that the weight map is smooth and free from noise.

The balance coefficient λ controls the trade-off between fidelity to the saliency map and smoothness of the weight map. The choice of λ is based on ablation experiments to achieve optimal performance.

(4) Fusion Strategy:

The final weight coefficients ω are computed as a weighted average of the optimized weight maps W_{ir} and W_{vis}. This ensures that the fusion process adaptively balances the contributions of the infrared and visible images based on their saliency information.

We have supplemented the aforementioned explanations in the method section. We hope this change meets your expectations. Thank you again for your constructive comments.

2. Elaborate on the selection method and basis of the regularization parameter in SVF and the balance coefficient in WLSO. It is feasible to compare the effects of different parameter values on the fusion results through experiments and provide recommended parameter ranges.

Response: We sincerely thank the reviewer for their valuable suggestion to elaborate on the selection method and basis of the regularization parameter φ in the SVF and the balance coefficient λ in the WLSO.

We appreciate the opportunity to provide a more detailed explanation of these parameters and their impact on the fusion results. In response to this suggestion, we have conducted additional experiments to analyze the effects of different parameter values on the fusion results. Below, we provide a detailed explanation of the selection method and basis for these parameters, along with recommended parameter ranges based on experimental validation.

(1) Regularization Parameter φ in SVF:

Through extensive experiments, we observed that the fusion results and quantitative metrics are relatively insensitive to variations in φ. Below, we provide a detailed analysis of this phenomenon based on the mathematical formulation of the SVF and the experimental results.

<1> **Mathematical Formulation**:

The weight coefficient C_k in the SVF is defined as:

$$C_k = \min\left(1, \frac{\sigma_{\max}^2}{\sigma_{\min}^2 + \varphi}\right) \quad (10)$$

Here, $\sigma_{\max}^2$ and $\sigma_{\min}^2$ are the maximum and minimum variance values in the filtering region WW, and φφ is the regularization parameter.

- When $\sigma_{\max}^2$ is significantly larger than $\sigma_{\min}^2$, the term $\frac{\sigma_{\max}^2}{\sigma_{\min}^2+\varphi}$ tends to be much greater than 1, regardless of the value of φ. In this case, C_k is clamped to 1, and the filter preserves the original pixel value I_k.
- When $\sigma_{\max}^2$ is close to $\sigma_{\min}^2$, the term $\frac{\sigma_{\max}^2}{\sigma_{\min}^2+\varphi}$ is close to 1, and C_k is also close to 1. In this case, the filter behavior is dominated by the local variance statistics rather than the value of φ.

As a result, φ has a limited impact on the filter behavior, especially when the local variance statistics exhibit significant differences.

<2> **Experimental Observations**:

We conducted experiments with different values of φ on the TNO datasets, the results of the quantitative comparison are shown in Table 1. The results showed that the fusion performance, as measured by metrics such as SF, MSE, SCD, and Qabf, remained relatively stable across different values of φ. This observation is consistent with the mathematical analysis, as the filter behavior is primarily determined by the local variance statistics rather than the regularization parameter φ.

Table 1 Fusion performance under different regularization parameter values φ

φ	SF↑	MSE↓	CC↑	SCD↑	SSIM↑	PSNR↑	Qabf↑
0.001	0.0261	0.0387	0.3594	1.9158	0.9556	62.2541	0.4744
0.05	0.0260	0.0388	0.3606	1.9176	0.9589	62.2431	0.4784
0.15	0.0260	0.0388	0.3606	1.9176	0.9588	62.2424	0.4784
5	0.0260	0.0388	0.3606	1.9176	0.9588	62.2421	0.4783

(2) Balance Coefficient λ in WLSO:

In addition, we conducted relevant ablation experiments on the choice of the balance coefficient λ in WLSO, aiming to optimize the performance of the proposed method. We performed the relevant experiments on the TNO dataset and the quantitative results are shown in Table 2. It can be found that, as λ increases, SF slightly decreases, indicating a reduction in high-frequency details. This is expected because a larger λ emphasizes smoothness in the weight map, which may suppress some fine details. MSE decreases as λ increases, indicating better fidelity to the source images. This is because a larger λ reduces noise and artifacts in the weight map, leading to more accurate fusion results. CC increases with λ, this is consistent with the improved fidelity observed in the MSE results. SCD decreases slightly as λ increases, indicating a more balanced integration of information from the source images. SSIM remains relatively stable across different values of λ, with a slight improvement at higher values. PSNR increases with λ, indicating better noise suppression and higher fidelity to the source images. Qabf increases slightly as λ increases, indicating better preservation of information from the source images. Based on the experimental results, we use λ =1 as the default value in this paper, as it provides a good balance between smoothness and fidelity, achieving high performance across multiple evaluation metrics.

Table 2 Fusion performance under different balance coefficient values λ

λ	SF↑	MSE↓	CC↑	SCD↑	SSIM↑	PSNR↑	Qabf↑
0.01	**0.0429**	0.0338	0.5526	**1.7484**	0.9256	62.8410	0.3971
0.1	0.0418	0.0336	0.5527	1.7478	**0.9257**	62.8627	0.3958
1	0.0405	0.0329	0.5532	1.7446	0.9236	62.9528	0.3971
3	0.0399	0.0322	0.5542	1.7412	0.9238	63.0507	0.3989
5	0.0396	0.0317	0.5550	1.7392	0.9242	63.1153	0.3999
7	0.0394	**0.0314**	**0.5558**	1.7377	0.9244	**63.1662**	**0.4000**

Thank you for your constructive feedback, and we hope the revised version meets your expectations.

3. The quantitative analysis indicators are not rich enough. We hope to add some evaluation indicators such as the message fusion quantity QAB/F. It should be noted that adding different evaluation indicators may lead to optimal conflicts, and we can truthfully explain the reasons.

Response: We sincerely thank the reviewer for their valuable suggestion to enrich the quantitative analysis by adding additional evaluation metrics, such as the message fusion quantity QAB/F [1].

We appreciate the opportunity to enhance the robustness and comprehensiveness of our evaluation framework. In response to this suggestion, we have conducted additional experiments and included the new evaluation metrics Qabf in our quantitative analysis.

The updated quantitative results are presented in Table 1, Table 2, and Table 3, along with a detailed discussion of the implications of these metrics. The relevant additions to the original manuscript are as follows:

4.2 Comparison experiments on the TNO dataset

"…The best SF and Qabf illustrates that our fusion results achieves the most refined sharpness, which is a direct result of our emphasis on preserving high-frequency details.…"

Table 1 Results of quantitative comparisons on the TNO dataset, where bold font indicates best and underlining indicates second best.

	SDNet	ICAFusion	GANMcC	SwinFusion	PIAFusion	TarDAL	TGFuse	CrossFuse	SeAFusion	Ours
Qabf ↑	0.2987	0.3014	0.3329	0.3991	0.4125	0.4239	0.3872	0.4019	<u>0.4689</u>	**0.4730**

4.3 Comparison experiments on the RoadScene dataset

"… .The second-ranked Qabf illustrates the advantages of our method in preserving scene edge features and reflecting information richness…"

Table 2 Quantitative comparison results on the RoadScene dataset.

	SDNet	ICAFusion	GANMcC	SwinFusion	PIAFusion	TarDAL	TGFuse	CrossFuse	SeAFusion	Ours
Qabf ↑	0.4398	0.4173	0.3972	0.5524	0.4419	0.3987	0.4331	0.4120	**0.5877**	<u>0.5791</u>

4.4 Comparison experiments on the LLVIP dataset

"…Attributed to the effective SVF-based decomposition and the visual saliency measure strategy based on WLSO, the proposed method effectively preserves the texture details while highlighting the salient information, as reflected in metrics SF, CC and Qabf… "

Table 3 Quantitative comparison results on the LLVIP dataset

	SDNet	ICAFusion	GANMcC	SwinFusion	PIAFusion	TarDAL	TGFuse	CrossFuse	SeAFusion	Ours
Qabf ↑	0.5329	0.5271	0.3895	0.5572	**0.6120**	0.2334	0.4968	0.5113	0.4732	0.6019

We believe that the inclusion of these additional metrics has significantly strengthened our quantitative analysis and provided a more comprehensive evaluation of our proposed method. Thank you for your constructive feedback, which has helped us improve the quality and rigor of our work.

Reference:

[1] Xydeas CS, Petrovic VS. Objective pixel-level image fusion performance measure. Proc SPIE 2000;4051:89 – 98.

4. The research motivation of the paper indicates that this method addresses the existing complex computational difficulties. However, the algorithm complexity analysis was not provided in the experimental section of this paper. (The running time can indirectly substitute for the complexity analysis.)

Response: We sincerely thank the reviewer for pointing out the need for an analysis of the computational complexity of our proposed method.

In response to this suggestion, we have added a detailed discussion of the algorithm's running time in the experimental section of the manuscript. We have supplemented relevant experiments in Section 4.6. The added content is as follows:

4.6 Analysis of Runtime Efficiency

Running efficiency is one of the important indicators for evaluating the performance of a method. Therefore, we conducted comparative experiments on running efficiency between our proposed method and nine advanced methods on the TNO dataset, and the results are listed in Table 7. It is evident that, due to the lightweight network architecture design and GPU acceleration, the deep learning-based method SeAFusion achieved a significant advantage. Additionally, CrossFuse ranked second. In contrast, our method, based on traditional optimization theory, requires extensive mathematical calculations to compute the optimal solution, and thus does not exhibit particularly outstanding performance in computational efficiency. Therefore, in future research, how to effectively reduce the iterative computation time will be an area that needs further exploration.

Table 7 Results of runtime comparison for 40 pairs of images on the TNO dataset. (Unit: seconds)

Method	SDNet	ICAFusion	GANMcC	SwinFusion	PIAFusion
Times	0.2013	0.2457	2.4002	1.9245	0.9351
Method	TarDAL	TGFuse	CrossFuse	SeAFusion	Ours
Times	1.2104	0.1722	0.1328	**0.0054**	1.9433

Thank you again for your constructive comments.

5. The references in the introduction section of the paper are too outdated. We hope to supplement or replace some with the latest literature, such as FusionOC, FusionPID, FusionCPP, etc. The more, the better.

Response: We thank the reviewers for their valuable suggestions.

Based on the reviewers' recommendations, we have added the following recent research papers and replaced some outdated ones in the introduction section.

[1] Dong L, Wang J. FusionOC: Research on optimal control method for infrared and visible light image fusion. Neural Networks, 2025, 181: 106811.
[2] Dong L, Wang J. FusionPID: A PID control system for the fusion of infrared and visible light images. Measurement, 2023, 217: 113015.
[3] Dong L, Wang J. FusionCPP: Cooperative fusion of infrared and visible light images based on PCNN and PID control systems. Optics and Lasers in Engineering, 2024, 172: 107821.

Responses to Reviewer 4's comments:

Reviewer #4: This paper presents an infrared and visible image fusion method based on sub-window variance filter and weighted least squares optimization. The studied topic is meaningful, and the proposed method seems reasonable. The authors may further improve the paper from the following aspects.

1. Some overview papers are suggested to be given for better referring of the relevant topics, for example visual quality and its modeling, e.g., 'Perceptual image quality assessment: a survey'; 'Perceptual video quality assessment: a survey'; 'Screen content quality assessment: overview, benchmark, and beyond'.

Response: We sincerely thank the reviewer for their valuable suggestion to include references to perceptual image quality assessment in our introduction. While the primary focus of our work is on image fusion, we recognize that perceptual image quality is an important aspect of evaluating fused images. Therefore, we have revised the introduction to explicitly mention the relevance of perceptual image quality assessment to our work and have added the related references [1][2][3]. These references provide a comprehensive overview of the field and help establish the connection between image fusion and perceptual quality evaluation.

The added content can be found in the first paragraph of the introduction:

"… . These techniques aim to enhance perceptual image quality by combining the strengths of both modalities, which is closely related to the field of perceptual image quality assessment [1][2][3]. …"

Thank you for your constructive feedback, which has improved the clarity and depth of our manuscript.

References:

[1] Zhai G, Min X. Perceptual image quality assessment: a survey. Science China Information Sciences, 2020, 63: 1-52.
[2] Min X, Duan H, Sun W, et al. Perceptual video quality assessment: A survey. Science China Information Sciences, 2024, 67(11): 211301.
[3] Min X, Gu K, Zhai G, et al. Screen content quality assessment: Overview, benchmark, and beyond. ACM Computing Surveys (CSUR), 2021, 54(9): 1-36.

2. Multimodal fusion as well as visual saliency are introduced in this paper, which have been involved in many studies (e.g., 'Study of subjective and objective quality assessment of audio-visual signals'; 'Fixation prediction through multimodal analysis'; 'A multimodal saliency model for videos with high audio-visual correspondence'). The authors may give some discussions on this aspect and the related works.

Response: We sincerely thank the reviewer for their valuable suggestion.

In accordance with the reviewers' suggestions, we have added a concise sentence that references the suggested studies [1][2][3], highlighting their contributions to multimodal analysis and saliency modeling. This addition establishes a clear connection between our work and the broader context of perceptual quality enhancement and multimodal data integration.

The added content can be found in the first paragraph of the introduction:

"… More importantly, recent advancements in multimodal fusion and visual saliency have demonstrated the importance of integrating complementary information and perceptual quality enhancement [1][2][3]. These studies provide valuable insights into the role of saliency-driven approaches and multimodal data integration, which inspire our work to improve the quality and interpretability of infrared and visible image fusion."

We hope this change meets your expectations. Thank you again for your constructive comments.

References:

[1] Min X, Zhai G, Zhou J, et al. Study of subjective and objective quality assessment of audio-visual signals. IEEE Transactions on Image Processing, 2020, 29: 6054-6068.
[2] Min X, Zhai G, Gu K, et al. Fixation prediction through multimodal analysis. ACM Transactions on Multimedia Computing, Communications, and Applications (TOMM), 2016, 13(1): 1-23.
[3] Min X, Zhai G, Zhou J, et al. A multimodal saliency model for videos with high audio-visual correspondence. IEEE Transactions on Image Processing, 2020, 29: 3805-3819.

3. In the literatures, besides quality metrics designed for image fusion quality assessment, there are also many general quality visual quality metrics, e.g., BPRI (Blind quality assessment based on pseudo-reference image), BMPRI (Blind image quality estimation via distortion aggravation), RichIQA (Exploring rich subjective quality information for image quality assessment in the wild), which are also suggested to reviewed and discussed.

Response: We sincerely thank the reviewer for their valuable suggestion to consider general visual quality metrics, such as BPRI, BMPRI, and RichIQA, for evaluating the quality of fused images. While these metrics provide important insights into perceptual quality assessment, they are primarily designed for general image quality evaluation rather than specifically for image fusion tasks. In the field of infrared and visible image fusion, it is common practice to use specialized metrics that are tailored to assess the unique characteristics of fused images, such as the preservation of salient features, texture details, and thermal information. These specialized metrics, including spatial frequency (SF), structural similarity (SSIM), and mutual information (MI) etc., have been widely adopted in the literature and are better suited to evaluate the performance of fusion algorithms in terms of their specific objectives.

That said, we acknowledge the potential value of exploring general quality metrics in future work to gain additional insights into the perceptual quality of fused images. We will consider incorporating these metrics in our subsequent studies to provide a more comprehensive evaluation framework.

Thank you for your constructive feedback, which has encouraged us to broaden our perspective on quality assessment in image fusion.
