# Supplementary material for: Saliency-enhanced infrared and visible image fusion via sub-window variance filter and weighted least squares optimization
Source: PLoS One. 2025 Jul 7;20(7):e0323285. doi: 10.1371/journal.pone.0323285 (PMC12233290; doi:10.1371/journal.pone.0323285)
Supplement: Attachment — Submitted filename: comments.docx [file pone.0323285.s001.docx]

The article was reviewed under the title "Infrared and visible image fusion method based on sub-window variance filter and weighted least squares optimization".

Overall, the focus of the study is good. However, I would like to offer several recommendations that authors may find useful in the process of revising their manuscript:

1- The abstract is very general, and it is necessary to provide more details in the abstract, given the variety of topics discussed.
2- Considering the classification of Related works into two categories: conventional methods and deep learning, it is better to examine more of the activities carried out in order to compare and show the strengths.
3- Considering the superiority of the model, if the challenges of previous research are examined, it can enrich the effectiveness of the article.
4- The article states that the weight factor (C_K_) is divided into 4 sub-windows. Are the 4 sub-windows specified by the authors or is it a fixed value?
5- If the authors chose 4 sub-windows, what was the reason for choosing this value?
6- Although the evaluation criteria used are well-known, it is better to include their full text in a footnote the first time their summary is used in the text of the article.
7- In the tables where the evaluation criteria obtained are compared with other studies, in some cases the criteria obtained by other researchers have a slight advantage, but the authors have stated that their model has superiority.
